# Using Gastrocnemius sEMG and Plasma α-Synuclein for the Prediction of Freezing of Gait in Parkinson's Disease Patients

**DOI:** 10.1371/journal.pone.0089353

**Published:** 2014-02-27

**Authors:** Xiao-Ying Wang, Wen-Yan Kang, Qiong Yang, Lin-Yuan Zhang, Sheng-Di Chen, Jun Liu

**Affiliations:** Department of Neurology & Institute of Neurology, Ruijin Hospital, Shanghai JiaoTong University School of Medicine, Shanghai, China; University of Melbourne, Australia

## Abstract

Freezing of gait (FOG) is a complicated gait disturbance in Parkinson's disease (PD) and a relevant subclinical predictor algorithm is lacking. The main purpose of this study is to explore the potential value of surface electromyograph (sEMG) and plasma α-synuclein levels as predictors of the FOG seen in PD. 21 PD patients and 15 normal controls were recruited. Motor function was evaluated using the Unified Parkinson's Disease Rating Scale (UPDRS) and Freezing of gait questionnaire (FOG-Q). Simultaneously, gait analysis was also performed using VICON capture system in PD patients and sEMG data was recorded as well. Total plasma α-synuclein was quantitatively assessed by Luminex assay in all participants. Recruited PD patients were classified into two groups: PD patients with FOG (PD+FOG) and without FOG (PD-FOG), based on clinical manifestation, the results of the FOG-Q and VICON capture system. PD+FOG patients displayed higher FOG-Q scores, decreased walking speed, smaller step length, smaller stride length and prolonged double support time compared to the PD-FOG in the gait trial. sEMG data indicated that gastrocnemius activity in PD+FOG patients was significantly reduced compared to PD-FOG patients. In addition, plasma α-synuclein levels were significantly decreased in the PD+FOG group compared to control group; however, no significant difference was found between the PD+FOG and PD-FOG groups. Our study revealed that gastrocnemius sEMG could be used to evaluate freezing gait in PD patients, while plasma α-synuclein might discriminate freezing of gait in PD patients from normal control, though no difference was found between the PD+FOG and PD-FOG groups.

## Introduction

Freezing of gait (FOG) is a paroxysmal locomotive gait disturbance observed in Parkinson's disease (PD) and is regarded as a disability phenomenon [1], [2]. The mechanism of FOG is considered to be multifactorial, with impairment of internal drivers as well as external factors, and is believed to be distinctly different from other parkinsonian features such as bradykinesia and rigidity [3]. The freezing of gait questionnaire (FOG-Q) was developed to reliably identify and screen out “Freezers” among PD patients [4]. Moreover, the identification of “Freezers” set the stage for application of kinematic analysis as an approach to unveiling the intrinsic characteristics of FOG [5]–[7]. However, a subclinical diagnostic test and prognosis marker for FOG in PD remains to be discovered.

Accumulating evidence has been shown that an absence or extreme reduction in particular muscles, including triceps surae (TS), tibialis anterior (TIA) and gastrocnemius (GAS), were observed in PD patients, a finding contrary to patients with ataxic gait [8]. TIA was thought to be the most affected muscle during the activation burst in late swing and had a relationship with lower UPDRS III total score [9]–[11]. In addition, Dietz et al. demonstrated that reduced GAS activity could contribute to shortening of the stride in PD [12]. Nieuwboer and her colleagues found a consistent pattern of premature activity of the TIA and GAS occurred before freezing, with the amplitude of TIA activity increased [5]. Although several results have been published, the characteristic muscle pattern activity in PD patients with of FOG remains controversial.

In addition to muscle activity patterns, investigators have studied the utility of putative biomarkers and their prognostic features and association with PD progression. Indeed, some PD-related biomarker candidates have been prospectively proven in clinical biomarker research, of which plasma α-synuclein was found to be a comparatively ideal biomarker and valuable in the diagnosis or monitoring of PD disease progression [13]–[16]. Our collaborator's study implied a trend of decreased plasma α-synuclein and DJ-1 levels in PD patients compared with healthy controls, but no statistical difference was reached [17]. Consequently, the important of plasma α-synuclein in discriminating different subtype of PD requires additional investigation.

Though α-synuclein was the most capital protein in the pathogenesis of PD, its relationship with motor function, especially gait disturbance in PD still remain unknown, several animal experiments had provided new clue to this point. Results were shown that rigidity and reduced locomotors activity were induced by the dual administration of α-synuclein oligomers plus fibrils into mice brain [18]. Another neuromuscular study revealed that α-synuclein might play a role in acetylcholine compartmentalization at the neuromuscular junction and in the fine control of activity of skeletal muscles [19]. When it comes to genetic research, *SNCA* variants were proved to be a strong predictors of faster motor decline in idiopathic PD, may help identify patients who will benefit from early intervention [20].

In our study, we recorded surface EMG (sEMG) data in PD patients to test the possibility of kinematics as a predictor of the freezing subtype of PD. In addition, we have also investigated the potential application of plasma α-synuclein as a discriminatory predictor of FOG in PD.

## Subjects and Methods

### 2.1 Study subjects

This study was approved by the Ethics Committee of Ruijin Hospital affiliated to Shanghai Jiaotong University School of Medicine. All participants provided their written informed consent to participate in this study. PD patients were recruited from the Department of Neurology, Ruijin Hospital affiliated to Shanghai Jiaotong University School of Medicine with a diagnosis of idiopathic PD based on the United Kingdom Brain Bank criteria. Patients were identified as having PD by at least two movement disorder specialists, of which 13 PD+FOG patients were identified by at least two movement disorder specialists not only based on Freezing of Gait Questionnaire (FOGQ), also identified by two criteria described as previous study [21]: (i)convincing subjective FOG reports, based on consistent and characteristic accounts of the phenomenon (including the typical feeling of the feet being glued to the floor, a hesitation or sudden block while initiating, walking or turning); (ii)a standardized and videotaped gait trajectory was performed containing specific elements known to provoke FOG [22]. These videos were rated offline for the presence of FOG by two different movement disorder experts. 8 PD-FOG patients matched for age, sex, disease duration, H-Y scale and UPDRS III score with PD+FOG patients, meanwhile, being willing to undergo gait trial were recruited. Participants were excluded if they had any other neurological disorders, previous orthopedic surgery or any musculoskeletal disorders that could affect gait. 15 healthy participants without Parkinsonism in their family history were also recruited (control group) from the Shanghai Wuliqiao community and were matched for age and sex with the disease groups. We assessed each PD patient with the Unified Parkinson's Disease Rating Scale (UPDRS) and Hohen-Yahr scale (H-Y scale) when they were in the “off” stage.

### 2.2 Gait questionnaire and analysis of PD patients

The FOG-Q was administered to evaluate the severity of freezing. The FOG-Q contains 6 questions, and a higher score indicates a more severe presentation of FOG. 21 patients volunteered to undergo gait analysis. Prior to gait analysis, all subjects were receiving pharmacological treatment with stable doses of antiparkinsonian medications and were tested while in the “on” stage. After 39 reflective markers were attached bilaterally to points of body surface projection [23], subjects were instructed to walk a 9-meter trajectory. The trial was initiated while the subjects stood barefoot in a relaxed position and a verbal command from the instructor was provided. Prior to data collection, the patients were instructed to practice the motions in order to maintain consistent performance. Data was collected using a ten-camera, 3-D motion capture system, with each subject performing a total of six gait trials.

sEMG data of the lower leg muscles (TIA and GAS) were collected bilaterally, using a portable integrated EMG module. After standard skin preparation, two electrodes were placed 1–2 cm apart on the belly of each muscle. EMG data were recorded at 1500 Hz. The signals were filtered at a low-pass cut-off frequency at 50 Hz. The data were then rectified at full-wave and smoothed by computing the root mean square of the signal using a window of 79 points. Mean amplitude EMG of all gait cycles (ampEMG, µV) and averaged area EMG of all gait cycles (areaEMG, µV*s) were recorded and calculated.

### 2.3 Blood plasma sample collection and contamination control

Blood samples (10 mL) were obtained from all patients and healthy controls by venous puncture between the hours of 8 a.m. and 10 a.m. Samples were collected in plastic tubes containing EDTA, and the plasma was then separated by centrifugation at 3000 rpm at 4°C for 20 min. Plasma was aliquoted into 0.2 ml plastic tubes and stored at –80°C. The samples were thawed on ice prior to analysis. The hemoglobin (HGB) levels and soluble P-Selectin (sP-Selectin) levels were measured as described in our collaborator's previous report to establish an index of the degree of red blood cell (RBC) and platelet contamination [17].

### 2.4 Magnetic bead-based Luminex assay of plasma α-synuclein in different groups

Magnetic COOH beads (Cat# MC10052-01, Bio-Rad, USA) were activated and coupled with antibody according to the manufacturer's protocol (Millipore, Billerica, MA, USA). Briefly, 100 µl beads were activated with 10 µl EDC (1-ethyl-3-[3-dimethylaminopropyl], 50 mg/ml) and Sulfo-NHS (N-hydroxy sulfosuccinimide, 50 mg/ml) in the ProteOn™ Amine Coupling Kit (Cat# 1762410, Bio-Rad, USA). 10–20 µg of mouse (monoclonal) anti-human α-synuclein antibody (Cat# AHB0261, Invitrogen, USA) was added to the activated beads with an incubation time of two hours. Then, the coupled beads were re-suspended in 150 µl of storage buffer, or an alternate storage buffer to complement the protein assay. Determination of bead concentration was performed using a Coulter Z2 counter or a hemocytometer to validate the efficiency of the coupling reaction. The wells containing the coupled beads were then covered with aluminum foil and stored at 4°C.

50 µl of capturing antibody-coupled beads (2,500 beads per well) were added to 96 well Bio-Plex Pro Flat Bottom Plates (Cat# 171025001, Bio-Rad, USA) and washed twice using the reagent kit (Cat# 171304071, Bio-Rad, USA). Then, 50 µl diluent recombinant human α-synuclein (Prospec, USA) was used as a standard, and plasma samples, diluted in equal sample dilution, were loaded on the magnetic plate with incubation for two hours at 1000 rpm on a plate shaker at room temperature in the dark, followed by washing the plate three times in succession. After incubation, detecting antibodies (2 µg/ml, biotinylated anti-human α-synuclein antibody, R&D systems, Minneapolis, MN, USA) were added at 50 µl per well on a rotator at room temperature for 60 minutes followed by washing three times, and the streptavidin-PE was diluted with assay buffer in the reagent kit for 30 minutes. The plate was then washed three times and each well received 125 µl assay buffer, followed by shaking for three minutes and then read via Liquichip Luminex 200™. The concentration of samples was calculated by comparison to a best-fit standard curve using a sigmoidal 5-parameter logistic equation. The recovery rate was close to 100% and coefficient of variation (CV%) in duplicate was less than 20%.

### 2.5 Statistical analysis

The demographic data and the results were analyzed using one-way analysis of variance (ANOVA) followed by the post-hoc Bonferroni test for comparison among three groups. T-test and Mann-Whitney U test were utilized for comparison between two disease groups. A *p*<0.05 was considered statistically significant. All statistic analyses were performed using Statistical Package for the Social Sciences (SPSS) 20.0 software.

## Results

### 3.1 Demographic characteristics and gait analysis

The demographic information of all participants is listed in **Table 1**. Participants with PD were further divided into two groups, those with freezing of gait (PD+FOG) and those without FOG (PD-FOG), based on a score of ≥2 on item three of the Freezing of Gait Questionnaire (FOG-Q), which indicates at least weekly freezing episodes. The average FOG-Q score was 14.46±4.41 in the PD+FOG group and 1.00±0.93 in PD-FOG group. In addition, H-Y scale (PD+FOG: 2.46±0.78, PD-FOG: 2.25±0.76), UPDRS III score (PD+FOG: 31.54±20.70, PD-FOG: 33.63±10.47), and the duration between the two disease groups were comparable. There were no statistically significant differences in age and sex among the three groups.

**Table 1:** Demographic information of different groups.

	PD+FOG (n = 13)	PD-FOG (n = 8)	Control (n = 15)
**Sex**	8/5	6/2	9/6
**Age (year)**			
Range	55–77	61–75	58–74
Mean	66.92±6.69	67.75±4.17	64.73±4.88
**H-Y scale**			
Range	1–4	1–3	-
Mean	2.46±0.78	2.25±0.76	-
**Duration (year)**			
Range	2–15	2–6	-
Mean	6.77±3.77	4.13±1.55	-
**UPDRS III score**			
Range	5–76	18–45	-
Mean	31.54±20.70	33.63±10.47	-
**FOG-Q score**			
Range	7–22	0–2	-
Mean	14.46±4.41	1.00±0.93	-

T-T test and one-way analysis of variance (ANOVA) were used for comparison between different groups. There were no statistically significant differences in demographic information among the three groups.

Abbreviation: PD+FOG = PD patients with freezing of gait; PD-FOG = PD patients without freezing of gait; H-Y scale = Hohen Yahr scale; UPDRS = Unified Parkinson's Disease Rating Scale; FOG-Q = Freezing of gait questionnaire.

The results of the gait trail in PD patients are shown in **Table 2**. We observed the following statistically significant parameters: slower walking speed (*p* = 0.006), smaller stride length (*p* = 0.004), smaller step length (*p* = 0.001) and prolonged double support time (*p* = 0.022) in the PD+FOG group compared to the PD-FOG group. However, there was no significant difference in other gait cycle parameters, including cadence, stride time, step time and step width between the PD+FOG and PD-FOG groups.

**Table 2:** Gait cycle parameters of PD+FOG group and PD-FOG group.

	PD+FOG	PD-FOG	*p* Value
Walking speed (m/s)	0.78±0.17	0.95±0.07	0.006
Cadence (step/min)	97.75±15.24	102.82±12.36	0.438
Stride length (m)	0.991±0.11	1.10±0.45	0.004
Stride time (s)	1.28±0.22	1.18±0.14	0.274
Step length (m)	0.50±0.06	0.58±0.02	0.001
Step time (s)	0.64±0.12	0.61±0.10	0.568
Step width (m)	0.15±0.03	0.13±0.04	0.305
Double Support (%)	25.19±3.12	22.09±7.84	0.022

T-T test and Mann-Whitney U test were utilized for comparing the gait cycle parameters between two disease groups. Compared to PD-FOG group, PD+FOG group displayed a decreased walking speed (*p* = 0.006), smaller stride length (*p* = 0.004), smaller step length (*p* = 0.001) and prolonged double support time (*p* = 0.022).

Abbreviation: PD+FOG = PD patients with freezing of gait; PD-FOG = PD patients without freezing of gait.

### 3.2 Surface EMG recording of PD patients

**Figure 1:**
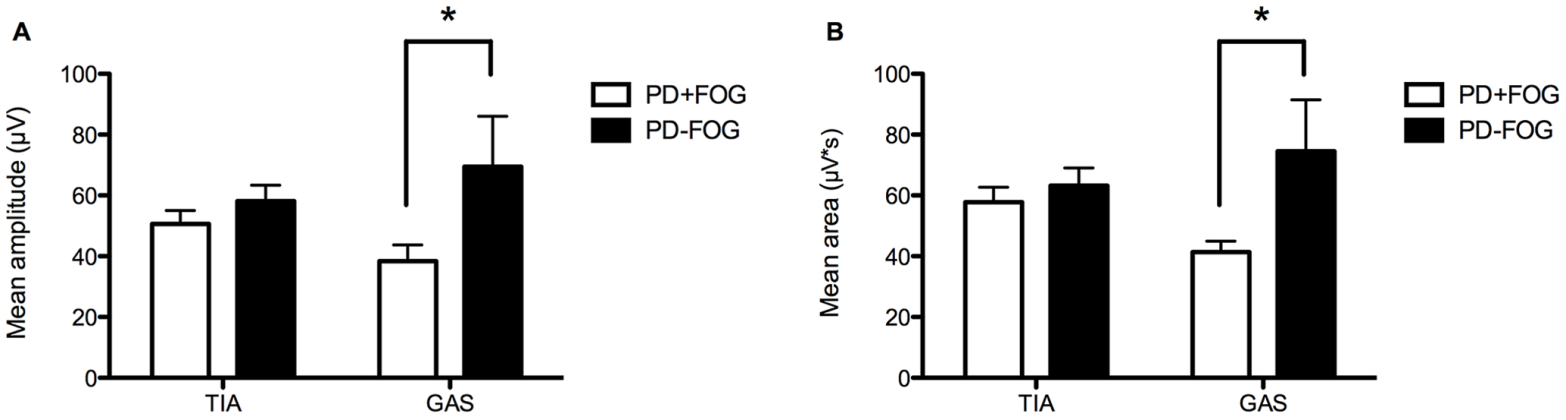
Surface EMG data of PD+FOG and PD-FOG group. (**A**) Mean amplitude of GAS activity was significantly reduced in the PD+FOG group compared to the PD-FOG group, but was similar for TIA activity. (**B**) Mean area of GAS activity was significantly reduced in the PD+FOG group compared to the PD-FOG group, but was similar for TIA activity.

In previous reports, the magnitude of both TIA and GAS activity was changed in PD patients. Our sEMG results showed that the mean amplitude and average area of GAS activity were lower in the PD+FOG group than the PD-FOG group (*p* = 0.046; *p* = 0.027) but not for TIA activity (*p* = 0.296; *p* = 0.498) (**Fig. 1A** **and** **Fig. 1B**), indicating that the GAS activity is related to freezing gait in PD. The raw EMG samples both before and after processed in different groups were seen **Figure S1**. Simultaneously, there was no significant difference in muscle activity of TIB and GAS between PD-FOG and Control group (**Figure S2**).

### 3.3 Plasma α-synuclein levels in different groups

The mechanistic etiology of FOG in PD patients is not clear. Some studies have shown that α-synuclein is involved in the pathogenesis of the locomotive impairment seen in PD, of which one study implied that in α-synuclein transgenic mice, robust sensorimotor impairments and a mild gait disorder develop at an early age [24]. To evaluate the value of plasma α-synuclein in the prediction of FOG in PD, we utilized a bead-based Luminex technique to measure the levels of plasma α-synuclein in different groups. To establish α-synuclein Luminex assay, the concentrations of samples in each plate were calculated according to each standard curve and dilution factors. The standard curve for the Luminex was obtained using the serially diluted recombinant human α-synuclein protein at different concentrations (**Figure S3**).

**Figure 2:**
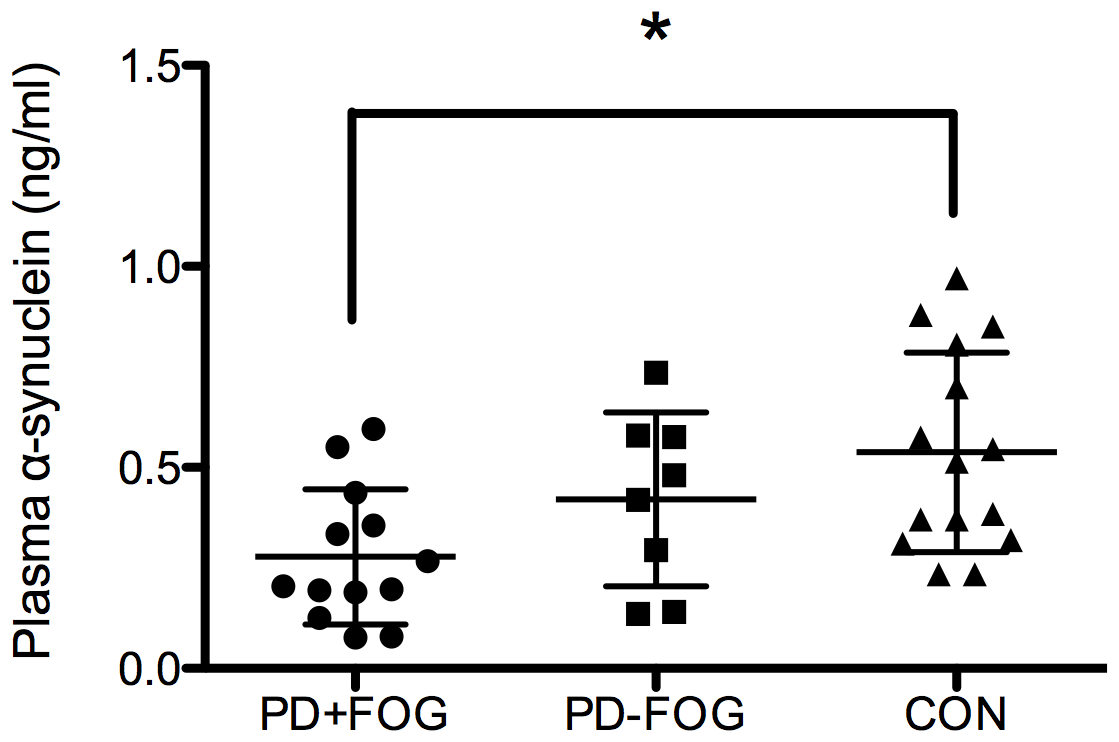
Plasma α-synuclein level of different groups. After controlling for blood contamination, plasma α-synuclein levels were decreased in the PD+FOG, PD-FOG and control groups successively. The difference between PD+FOG and control group achieved significance but there was no statistical difference between the PD-FOG and control groups.

The results demonstrated that the level of plasma α-synuclein in the PD+FOG group was significantly decreased compared to the control group (*p* = 0.009) (**Fig. 2**). However, there was no significant difference between the control group, PD+FOG group and PD-FOG group, respectively (*p* = 0.666, *p* = 0.448) (**Fig. 2**).

## Discussion

In the present study, we have explored the diagnostic and prognostic value of plasma α-synuclein levels in PD and a PD-related gait disorder referred to as FOG. Moreover, we have also described the characteristics of the gait cycle and magnitude of muscle activity in PD patients, providing information on the potential value of sEMG as a predictive tool and the neurological mechanisms in FOG of PD.

Gait analysis of PD+FOG patients demonstrated the typical gait characteristics, including: slowed walking speed, reduced stride length and step length, and prolonged double support time. One study demonstrated that both reduced step length and sequence effect could contribute to freezing [6]. Although the gait performance of PD patients could be well described using a three-dimensional motion system, the responsible structure related to the pathological mechanism of gait deficits remains unknown.

There is a paucity of research regarding the mechanistic etiology of motor impairment, especially in PD gait disorders. Accumulating evidence indicates that α-synuclein levels in body fluids have great potential for becoming a diagnostic biomarker of PD, even in discriminating between Lewy-body-related dementia and AD [15]. Whether its level is related to locomotive impairment in PD remains to be investigated. Transgenic mice studies have demonstrated that overexpression of α-synuclein results in early sensorimotor impairment [24]. In this study, we found that plasma α-synuclein levels were helpful in differentiating PD patients with FOG from normal people, though there was no difference between the PD+FOG and PD-FOG groups.

Simultaneously, we have observed a significantly reduced activity in the GAS of patients in the PD+FOG group versus patients in the PD-FOG group, which indicates that GAS activity might be used as a potential clinical predictor in discriminating freezing type in PD. This result is consistent with a previous report that poor recruitment of the GAS was much more apparent in parkinsonian patients with FOG compared to those without FOG [25], supporting the hypothesis that the synergistic effect of deficits in magnitude and timing of stepping were fundamental to freezing gait [5]. In our study, abnormalities muscle sEMG patterns were also compatible with reports that the low speed movements of PD patients are attributable to reduced EMG activity and simplified neuromuscular control [26], [27].

In summary, plasma α-synuclein was found to be valuable in distinguishing freezing type in PD from normal control, although its level was not changed in among the different PD groups. The GAS muscle activity was reduced in PD patients with FOG, indicating that sEMG could be used in quantifying freezing of gait in PD patients. However, future studies will require a larger cohort in order to validate this finding.

## Supporting Information

Figure S1 **The raw EMG samples both before and after processed in groups.** EMG data of different groups was recorded at 1500 Hz. The raw signals were low-pass filtered at a cut-off 50 Hz, followed by full-wave being rectified and smoothed by computing the root mean square of signals. The EMG signals before and after processed of three subjects in PD+FOG, PD-FOG and Control group were shown in **Figure S1** **A**, **B**, and **C**, respectively.
(TIF)

Figure S2 **The comparison between PD-FOG and Control group in surface EMG data.** To demonstrate whether surface EMG can be used as a unique marker to FOG rather than PD, the EMG data of TIB and GAS was also recorded in healthy controls. The average age of subjects was 64.29±3.55 (ranged from 58–68), which was similar to that of PD+FOG and PD-FOG group. The EMG data was compared between PD-FOG and Control group (CON). As shown in **Figure S2**, there was no significant difference in muscle activity of TIB and GAS between PD-FOG and Control group.
(TIF)

Figure S3 **The standard curve of established α-synuclein Luminex assay.** To establish α-synuclein Luminex assay, the concentrations of samples in each plate were calculated according to each standard curve and dilution factors. The standard curve for the Luminex was obtained using the serially diluted recombinant human α-synuclein protein at 2, 1, 0.5, 0.25, 0.13, 0.06, 0.03 and 0.02 ng/mL. Assay was performed to select the best mathematical model for curve fitting and the dilutions.
(TIF)
