# Supplementary figures and images for: Using Gastrocnemius sEMG and Plasma α-Synuclein for the Prediction of Freezing of Gait in Parkinson's Disease Patients
Source: PLoS One. 2014 Feb 27;9(2):e89353. doi: 10.1371/journal.pone.0089353 (PMC3937335; doi:10.1371/journal.pone.0089353)

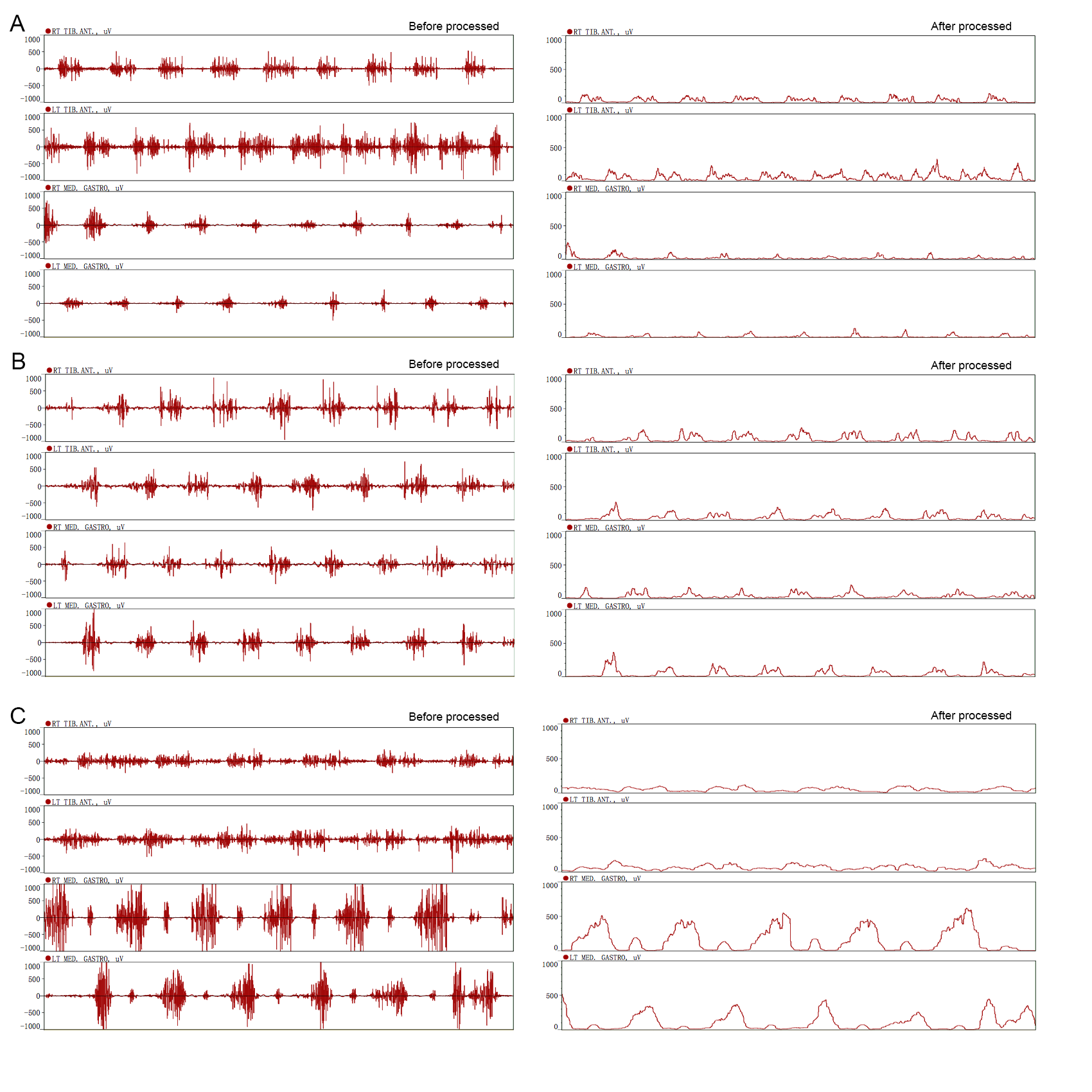

Supplement: Figure S1 — The raw EMG samples both before and after processed in groups. EMG data of different groups was recorded at 1500 Hz. The raw signals were low-pass filtered at a cut-off 50 Hz, followed by full-wave being rectified and smoothed by computing the root mean square of signals. The EMG signals before and after processed of three subjects in PD+FOG, PD-FOG and Control group were shown in Figure S1 A, B, and C, respectively. (TIF) [file pone.0089353.s001.tif]

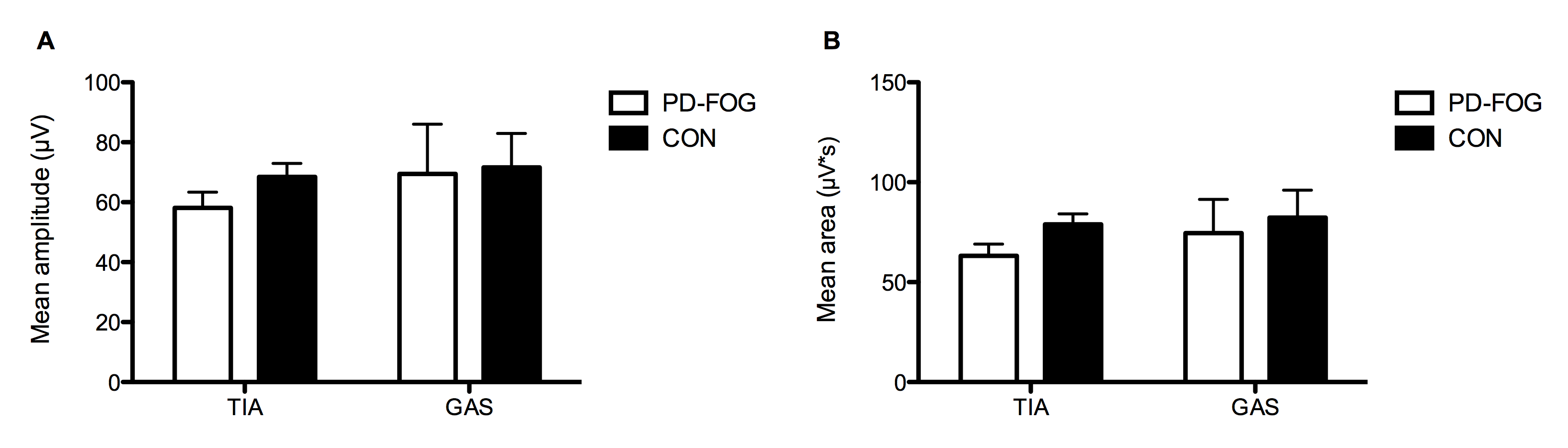

Supplement: Figure S2 — The comparison between PD-FOG and Control group in surface EMG data. To demonstrate whether surface EMG can be used as a unique marker to FOG rather than PD, the EMG data of TIB and GAS was also recorded in healthy controls. The average age of subjects was 64.29±3.55 (ranged from 58–68), which was similar to that of PD+FOG and PD-FOG group. The EMG data was compared between PD-FOG and Control group (CON). As shown in Figure S2, there was no significant difference in muscle activity of TIB and GAS between PD-FOG and Control group. (TIF) [file pone.0089353.s002.tif]

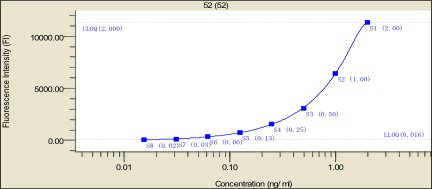

Supplement: Figure S3 — The standard curve of established α-synuclein Luminex assay. To establish α-synuclein Luminex assay, the concentrations of samples in each plate were calculated according to each standard curve and dilution factors. The standard curve for the Luminex was obtained using the serially diluted recombinant human α-synuclein protein at 2, 1, 0.5, 0.25, 0.13, 0.06, 0.03 and 0.02 ng/mL. Assay was performed to select the best mathematical model for curve fitting and the dilutions. (TIF) [file pone.0089353.s003.tif]
